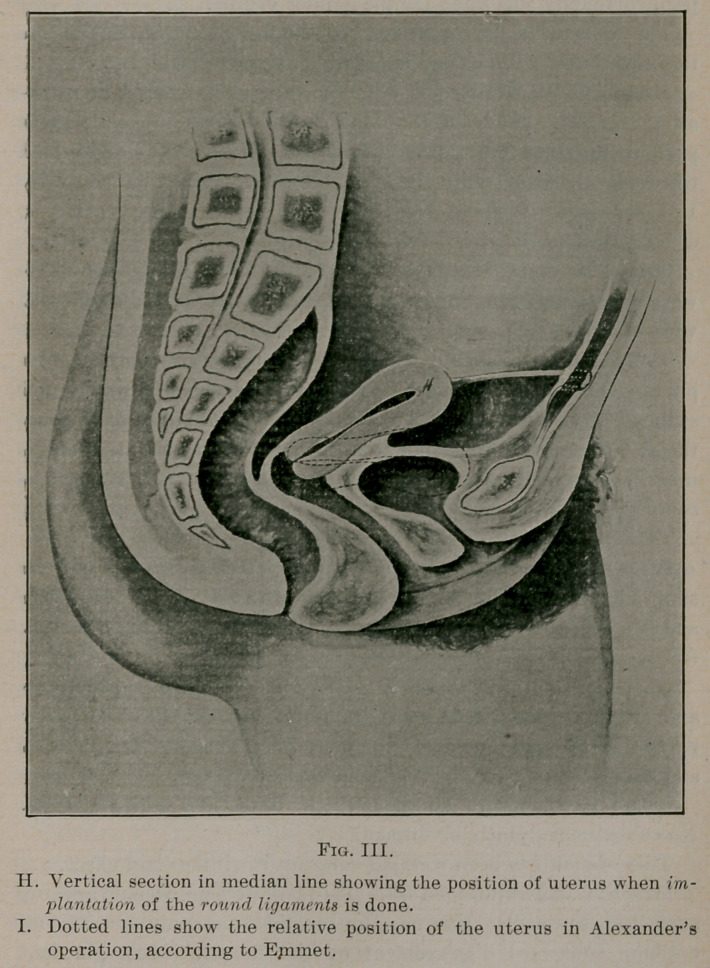# Suspension of the Uterus by Intramural Shortening of the Round Ligaments

**Published:** 1899-10

**Authors:** Geo. H. Noble

**Affiliations:** Atlanta, Ga.


### SUSPENSION OF THE UTERUS BY INTRAMURAL SHORTENING OF THE ROUND LIGAMENTS.

BY GEO. H. NOBLE, M.D.,

ATLANTA, GA.

The uniform attainment of satisfactory results in reposition and fixation of downward and backward displacements of the uterus fail for reasons that the anatomical supports utilized are either too weak or are imperfectly fixed. In ventro-suspension, the pedicle often acts as an irritant on account of the buried suture in the fundus, encouraging hyperemia of the uterus, and frequently interferes seriously with the development of the uterus in the process of gestation, or stretches away from the abdominal parietes and becomes useless.

With the hope of overcoming these, and minor disturbances, the writer began the study of the question some years ago, and after devising the method here presented delayed, in operating, endeavoring to thoroughly consider both sides of the question, lest some objectionable feature might defeat the object to be attained. Finding no serious obstacle, it was put to the first test April 6th, 1893, in the Grady Hospital.

The operation was carried out in detail, as it had been planned and, much to the gratification of the writer, has proven perfectly successful, lasting uninjured for the past six and a half years, under the strain usual to advanced procidentia uteri. Desiring to await secondary or deferred results, rather than to proceed unguardedly with a multiplicity of cases, ample time was allowed before doing the second case, which was operated upon December 8th, 1895. The others occurred in the following order: July 9th, 1896; March 21st, 1897; January, 1898. The records of the hospital show the details of these cases. In addition to the foregoing I have operated seven times in my private infirmary (see below).

All cases done up to date have proven perfectly satisfactory excepting an infected suture in one case, which suppurated and required removal. The infection, caused by accidental slipping of the dressing, extended to the buried suture on the left side but did not prevent satisfactory suspension of the uterus.

The operation is an intramural and extraperitoneal implantation of the round ligaments.

The first step consists in making the usual abdominal incision in the median line, for the purpose of exploring the pelvic cavity, breaking up adhesions, removing diseased appendages, etc.

Second. After thorough inspection, the round ligament on the side most convenient is seized with a pair of light compression forceps with teeth in end (Senn's) and drawn toward the center of the pelvic cavity, with a view of rendering the distal end of the ligament taut to facilitate manipulations outside the peritoneum. A pair of artery forceps is then passed through the posterior sheath of the rectus muscle at its outer border, and at a point an inch to an inch and a half above a line drawn from one internal abdominal ring to the other. The forceps are spread open and withdrawn thus, making an aperture sufficiently large to admit the finger easily. Through this opening the finger is passed behind* the peritoneum to the tightly drawn, cord-like ligament, which is in the direction of the internal opening of the inguinal canal. By a succession of downward movements, the peritoneum is stripped from it for an inch or two.

* Extraperitoneal.

Third. The forceps are here given to an assistant to hold while the operator passes a similar pair above the finger in the subperitoneal fatty tissue and grasps the round ligament as near the peritoneum as possible, and draws it out as far as its attachments will permit.

FIG. I.

A. Forceps in peritoneal cavity grasping *round ligament* and drawing the same in the direction of the center of the pelvic cavity, to render tense the distal end and to drag the peritoneum away from the internal abdominal ring.

B. Forceps passed along the finger in the subperitoneal fatty tissue to the *round ligament*, for the purpose of drawing a loop of the same between the *rectus muscle* (c) and the *transversalis fascia* (d).

Fourth. The stripping is again resumed, and when sufficient another pair of forceps is introduced below the second, and an additional section of the ligament drawn up, the other forceps being removed. The stripping continues from the support given by the forceps in the hand of the operator, until the ligament is drawn well out of the wound, between the rectus muscle and transversalis fascia, to the edge of the incision. The process is then repeated upon the other side.

Fifth. Both ligaments are clamped together in the median line, which places the uterus near, or about the position where it is to be fixed. They are carefully freed from fat or foreign tissue, in order to permit direct contact and adhesion to the recti muscles and transversalis fascia.

FIG. II.

E. The loop of *round ligament* drawn between the *rectus muscle* and the *transversalis fascia,* and anchored to same by *buried suture* (f).

G. *Buried suture* uniting the extremities of the loop in the median line.

Sixth. Then on each side where the ligament appears through the opening made in the posterior sheath of the rectus muscle, *i. e.,* the outer border, a silver or silk suture threaded upon a stout curved needle is passed through the transversalis fascia from within outward, and on returning takes about half an inch of the fascia in the stitch. It is then carried through the round ligament, rectus muscle and posterior sheath, and returned through sheath, muscle and ligament, taking in about three-fourths of an inch in this stitch. The sutures are tied carefully but not tightly, cut or twisted, and the ends of the wires folded under twice, to prevent sticking of the sharp points.

Seventh. The ligaments are released at the median line, where another suture is passed, taking fascia, ligament, muscle and sheath on one side, and in the reverse order on the opposite side, and twisted or tied upon the external surface of the fascia. It is better not to fasten this stitch until the abdominal sutures are in.

Eighth. Close with silkworm-gut, and apply aseptic dressings, the edges of which should be glued to the abdomen with flexible collodion. No pessary will be required unless the uterus is unusually heavy.

The ligaments have been drawn between the rectus and fascia, for the purpose of contracting extensive adhesion to the unyielding structures, which becomes sufficiently strong to support the uterus after disintegration or absorption of the suture takes place, and to secure direct suturing to the transversalis fascia, a tissue possessing strength and elasticity, elements that are necessary to ideal implantation or anchorizing of a support upon which more or less constant weight and frequent impulse of intra-abdominal pressure are exerted. Supports receiving their attachments from fixed points, such as bony structure or fascia in close proximity to the same, receive the brunt of the force more or less suddenly, and in consequence participate in the strain, with a degree of injury, each time the force is applied. Per contra, supports receiving attachment from a firm, elastic structure, such as the transversalis fascia, at a point distant from its bony attachment, do not feel the sudden impulse of the force above mentioned, but receive it gradually as it is expanded upon the elastic parietes of the abdomen.

The danger of the operation is about on a par with an exploratory incision, so far as the abdominal cavity is concerned. The separation of the peritoneum is not a serious question. Large areas of it may be stripped off with impunity, especially when there are three sides of the field attached, as in this case. It will not pouch, for the intra-abdominal pressure keeps it in close relation to the parietes.

There is no danger of injuring the epigastric vessels, as the tension made upon the ligament, draws it and the peritoneum sufficiently far away from the internal abdominal ring, to render the manipulation safe.

This operation possesses many advantages over others designed for the same purpose. There is but one incision; through it the pelvic complications are accessible.

The point of attachment is nearer the uterus, where the round ligament is larger and stronger, and therefore it does not depend upon the weak or distal end of the ligament for support, as the operations of Wilie, Emil Bordé, Mann and others do.

It is applicable to a greater range of cases.

It does not interfere with physiological mobility of the uterus, or its development in pregnancy.

No bladder irritation or dysuria is caused by it.

There are no sutures in the fundus to irritate the uterus.

To all peritoneal attachments, it is superior in permanency. It cannot stretch away or pull off, as the ligament is fixed to the transversalis fascia by a buried suture.

It is an extra-peritoneal fixation, and leaves no points of irritation to contract adhesions to the abdominal viscera.

It does not prolapse or antevert the uterus as Emmet claims the Alexander operation does, but suspends it in a more perpendicular position, that is, in the axis of the brim of the pelvis.

Mrs. A. M. M., aged 46. Small, anemic, poorly nourished women, six children, menstruation free, regular, aching, etc. Suffering from complete procidentia uteri, entire uterus lying outside of the vulva. Perineum lacerated to the sphincter ani muscle. Vaginal orifice very much dilated. Muscles of perineum relaxed and atrophied from disuse.

Abdomen opened April 6th, 1893. No adhesions, appendages healthy, but displaced, etc. Round ligaments drawn up and implanted between recti muscles and transversalis fascia with No. 24 silver wire (as described).

The result was very favorable, scarcely any elevation in temperature or pulse curves. No harm came from the rather free separation of peritoneum from abdomen, which was more extensive than what occurred in subsequent cases.

FIG. III.

H. Vertical section in median line showing the position of uterus when *implantation* of the *round ligaments* is done.

I. Dotted lines show the relative position of the uterus in Alexander's operation, according to Emmet.

Abdominal suture removed at the end of the seventh day; out of bed on the fifteenth day. The uterus remains in an elevated position (i. e., in the axis of the brim of the pelvis), freely movable, and creates no discomfort. Six weeks subsequently colpo-perineorrhaphy was done for the gaping vagina.

This woman has had hard domestic duties that have put the case to a severe test without any unfavorable signs resulting.

Mrs. E. C. W., white, aged 35. Anemic, neurasthenic, menstruating regularly, but scantily. Had been confined to bed a great part of the time since last childbirth, two years since. Had retroversion, with subinvolution. Uterus increased in size fully 100 per cent. Suffered bearing down pains, dragging sensations, dysuria, and an occasional attack of cystitis.

Operation done December 8th, 1895. The round ligaments were drawn between the recti muscles and fascia, and fixed with wire sutures.

There was scarcely any elevation of temperature or change in pulse. Results satisfactory. The uterus remains elevated and swings freely. The round ligaments were not attached as high up the abdomen as recommended, or as in the other cases, so the fundus uteri is lower, or nearer the bladder, though not near enough to cause pain or discomfort.

This case was out of bed on the 24th of December.

N. J., yellow woman, aged 35. No children. Has had chronic subinvolution and muco-purulent discharge following miscarriage and an attack of pelvic peritonitis. Had been wearing a pessary for five years.

Operation July 9th, 1896. Many adhesions of appendages and uterus to intestines and omentum, but delicate and easily ruptured. Round ligaments implanted between the recti muscles and transversalis fascia by silver wire suture.

Result satisfactory. Uterus swings freely, at a high elevation. No subsequent dysuria or discomfort.

This women has been cooking constantly without bad effect.

Mrs. F. C. S., white, aged 36. Tall, large-frame woman, anemic, syphilitic (tertiary). Has been an invalid from subinvolution and extreme backward displacement of the. uterus. Muco-purulent discharge, cyst of the left ovary.

This woman has been under my observation for two years.

Has been relieved very greatly of the anemia; treatment for the local or pelvic diseases with much relief; also under constant treatment for syphilis.

Operation done March 21st, 1897. Cyst of left ovary, size of an egg, removed; numerous adhesions broken up. Round ligaments implanted between recti muscles and fascia, with silver wire.

Recovery slow, but good. No unfavorable symptoms. The uterus remains high and freely movable. No bladder symptoms.

L. T., of Florence Crittenden Home, aged 24. Gave history of normal confinement two years ago. Cervix-uteri lacerated on one side. Uterus subinvoluted, retroverted, and lying low in pelvis. Frequent attacks of hysteria, disposed to complain of trivial matters. Frequent dysuria, pain at times said to be severe.

Operation done January 20th, 1898. Adhesions universal, but not very dense—separated on slight pressure. Round ligaments drawn between the recti muscles and fascia at a point a little higher than any other case, drawing uterus up more than necessary, so that for a time she complained of pulling sensations at the points where the ligaments were attached.

The case made a good recovery, excepting that the lower end of the dressing became loosened, the wound uncovered, and infection of the two lower stitches occurred on the fifth day, resulting in cellular abscess, about the size of an egg, on the left side. It was promptly drained and healed without delay.

November 8, 1898. Mrs. H., double salpingitis, cysts of each ovary, extensive pelvic adhesions, uterus bound down in pelvis by very dense adhesions. Appendage on both sides removed, uterus suspended by intramural shortening of round ligaments. Silk suture. Cured.

January 17, 1899. Miss B., hystero-mania, uterus retroverted, hyperesthetic, could not bear pessary, uterus engorged and covered by numerous bands of delicate adhesions, very anemic. Suspended by intramural shortening of round ligaments. Silk suture. Success. Mania and hyperesthesia also relieved.

February 11, 1899. Mrs. S., old pyosalpingitis double; pelvic organs deeply imbedded in dense adhesions, slight tear in perineum; hystero-epilepsy, very anemic and neurasthenic, double salpingo-oophorectomy. Silk suture. Cured.

March 19, 1899. Mrs. H., cyst size of an egg of L. ovary, subinvolution, retroversion, extensive adhesions, mitral stenosis; very anemic. Cyst removed, uterus suspended by intramural shortening of round ligaments. Result: Relief of displacement and a great deal of pelvic discomfort, but has not entirely recovered from anemia and neurasthenia. Silk suture.

April 12, 1899. Mrs. J., retroversion and prolapsus to vaginal outlet, anemia, hysteria marked, partial loss of perineum. Intramural shortening of round ligaments. Displacement corrected. Anemia and nervous symptoms much improved at time of dismissal. Silk suture.

June 3, 1899. Mrs. S., subinvolution, retroversion, fibroid tumor size of walnut on fundus uteri, extensive adhesions, anemic and neurasthenic, lacerated perineum. Suspension of uterus by intramural shortening of round ligaments, removal of fibroid tumor by excision. Perineorrhaphy. Silk suture. Cured.

June 6, 1899. Mrs. A., anemia, lacerated perineum, both kidneys at crest of ileum, uterus retroverted and firmly fixed in concavity of sacrum. Intramural shortening of round ligaments. Perineorrhaphy. Relieved except some slight discomfort from dragging kidneys. Silk suture.

---

### ATROPHY OF THE SEXUAL ORGANS.

I have had some experience in this trouble and have found wonderful success from the following formula in cases where masturbation has been and is practiced:

℞ Tinct. sepia officinalis.................................ʒ 5.

Tinct. pulsatilla.........................................ʒ 11.

M. Sig.: Ten to fifteen drops four times a day, the last dose on going to bed.

By getting your patient to exercise a little will-power you will find no trouble in effecting a cure. In all cases where the sexual organs are abused by overuse the cerebellum becomes diseased, and consequently your patient loses all power of self-control. Cure the disease and the habit will cure itself.—*Thos. A. E. Evans, M.D., Farmers, Ky., in Medical Brief.*

## Figures and Tables

**Fig. I. f1:**
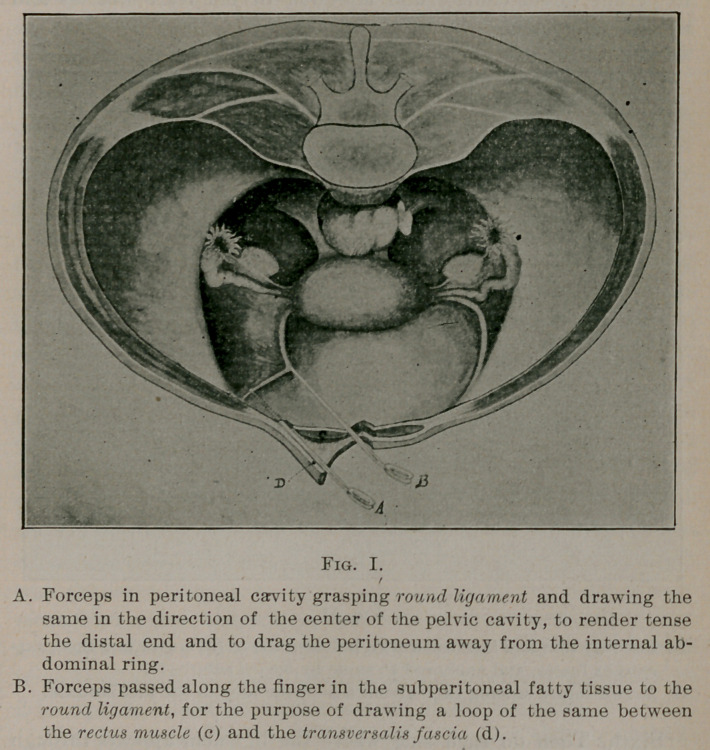


**Fig. II. f2:**
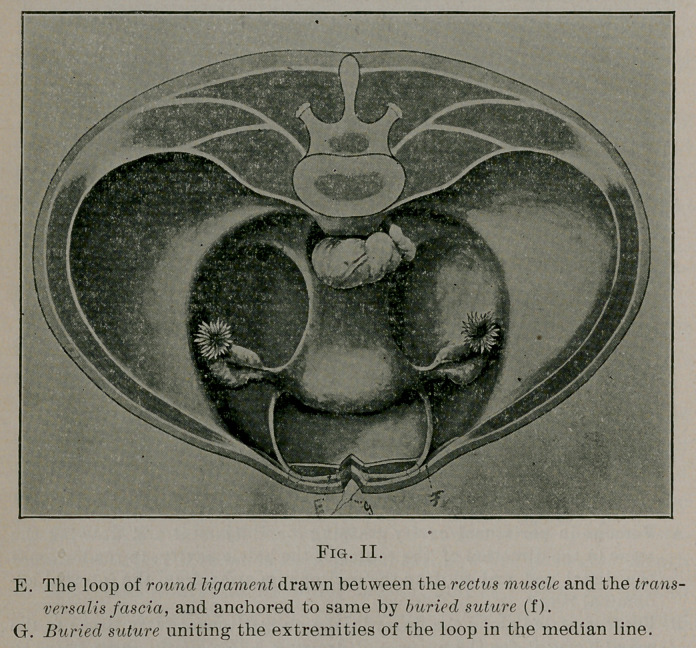


**Fig. III. f3:**